# Effect of Intraoperative Sublingual Nitroglycerin on the Rate of Active Uterine Segment Incision in Preterm Cesarean Birth

**DOI:** 10.7759/cureus.101471

**Published:** 2026-01-13

**Authors:** Judy Jiang, Alexandra Berra, Anna Rybinska-Campbell, Brittany Butler, Megan Breen, Angela C Ranzini

**Affiliations:** 1 Obstetrics and Gynecology, MetroHealth Medical Center/Case Western Reserve University School of Medicine, Cleveland, USA; 2 Obstetrics and Gynecology, MetroHealth Medical Center, Cleveland, USA; 3 Obstetrics and Gynecology, Saint Peter’s University Hospital, New Brunswick, USA

**Keywords:** cesarean section (cs), lower uterine segment, maternal blood loss, nitroglycerin (ntg), preterm birth (pb)

## Abstract

Introduction and aim: Active segment uterine incisions, including classical, T-shaped, and high transverse incisions, are often performed during preterm cesarean deliveries (CD), especially in patients delivering at less than 30 weeks of gestation, when the lower segment can have inadequate space for a safe maternal or fetal delivery. These uterine incisions increase the risk of maternal morbidity due to longer operative time, increased blood loss, and the rate of uterine dehiscence in future pregnancies compared to lower segment uterine incisions. Patients with uterine incisions in the active segment are also not candidates for future trials of labor. Nitroglycerin is known to cause uterine relaxation and has been used in cases of uterine inversion, manual removal of the placenta, difficult fetal extractions at cesarean delivery, and external or internal version for second twins. This study aimed to determine if intraoperative sublingual nitroglycerin is associated with a decreased active segment uterine incision rate in patients undergoing CD prior to 30 weeks of gestation. The outcomes included maternal blood loss, umbilical artery pH, and 1-min and 5-min Apgar scores.

Study design: In this retrospective case-control study, patients with singleton pregnancies between 22 weeks 0 days and 29 weeks six days of gestation undergoing CD at two community-based hospitals were compared between the years of 2017 and 2023. Maternal and neonatal variables were collected and analyzed using equality of means and Mann-Whitney U tests as appropriate.

Results: Patients who received nitroglycerin (n=26) were compared with patients at the same week of gestation who did not receive nitroglycerin (n=26) during cesarean delivery. The rate of active segment uterine incision was lower in the group that received intraoperative nitroglycerin (26.9%) compared to the group that did not (61.5%; p=0.012). Quantitative blood loss (QBL) was significantly decreased in patients who received nitroglycerin, with a mean QBL of 583 mL compared with 944 mL in patients who did not (p=0.031). Neonatal outcomes, including umbilical artery pH and Apgar scores, were not significantly different between the two groups.

Conclusion: Intraoperative nitroglycerin administration was associated with a significantly lower rate of active segment uterine incision in patients undergoing CD and decreased maternal blood loss. Neonatal outcomes were similar between groups.

## Introduction

Preterm cesarean deliveries increase the risk of cesarean section with a uterine incision through the active segment, including classical cesarean section, in comparison to full-term cesarean deliveries [1]. These uterine incisions increase the risk of maternal morbidity with longer operative time, increased blood loss, and increased rate of uterine dehiscence and rupture in future pregnancies compared to lower segment uterine incisions, and preclude vaginal delivery in subsequent pregnancies [1,2].

Data from human and experimental animal studies indicate that nitroglycerin (NTG) can effectively cause relaxation of the uterus [3]. Nitric oxide, a novel messenger formed during the nitric oxide synthase-catalyzed oxidation of L-arginine to L-citrulline, is involved in maintaining normal uterine tone during gestation [4]. NTG can be given sublingually, intravenously, intranasally, or transdermally. It has a clinically evident onset of action between 30 and 45 s and a short half-life of between 2 and 3 min [5]. The sublingual preparation has a long shelf life and is quick and easy to administer, making it an ideal medication to temporarily relax the uterus [6].

In obstetrics, nitroglycerin has been used to cause temporary uterine relaxation to facilitate fetal extraction at cesarean section with malpresentation or head entrapment, external or internal version for second twins, management of retained placenta with a contracted cervix, and management of uterine inversion without apparent maternal adverse effects [7-10]. However, some have suggested that there may be an increased risk of hemorrhage and uterine extension [9]. In addition, though data are limited, no significant fetal side effects of nitroglycerin use have been noted [4,7,8].

We evaluated the use of nitroglycerin to reduce the rate of active segment incisions by helping to relax the lower uterine segment, thereby allowing widening of the lower segment in order to achieve a safe lower segment incision in patients having cesarean deliveries between 22 and 29 weeks of gestation.

This article was previously presented as a meeting abstract at the 2025 Society for Maternal Fetal Medicine Pregnancy Meeting on January 31, 2025.

## Materials and methods

A retrospective case-control study of patients delivering at two community-based hospitals, the MetroHealth System (MH) and Saint Peter’s University Hospital (SPUH), between January 1, 2017, and December 31, 2023, was performed. Inclusion criteria were patients with singleton pregnancies between 22 weeks 0 days and 29 weeks six days of gestation undergoing cesarean delivery (CD) at SPUH who were matched to controls who had cesarean deliveries on the same day of gestation from MH. Data were collected using the Epic Medical Record Systems (MH)(Verona, Wisconsin: Epic Systems Corp.) and the Paragon EMR (SPUH) (Chicago, IL: Allscripts). Maternal age, race/ethnicity, insurance status, gestational age at birth, quantitative blood loss, neonatal arterial blood gas, and neonatal Apgar scores were collected. Post-hoc power analysis using GPower 3.1 (Düsseldorf, Germany: Heinrich Heine University Düsseldorf) determined insufficient power to detect differences in Apgar scores (power=0.16 and power=0.34 at p<0.05) and for cord pH (power=0.22 at p<0.05), but confirmed that the sample is sufficient to power statistical analysis of differences in low transverse incision and quantitative blood loss (QBL) (power>0.80 at p<0.05) [11].

The standard of care at Saint Peter's University Hospital was for the delivering team to evaluate the uterus to decide if nitroglycerin would be indicated during the cesarean section. This would typically be done on evaluation of the uterus prior to hysterotomy intraoperatively. Patients who received nitroglycerin received two sprays of 400 μg (800 μg total) of nitroglycerin under the tongue intraoperatively immediately prior to the hysterotomy. After uterine relaxation was observed, a final decision to make either a low transverse or active segment uterine incision was made by the delivery team based on the perceived distance between the vessels and sidewalls of the uterus. Once the baby was delivered, the placenta was removed just after the uterus began to contract. Quantitative blood loss was calculated after each delivery. Patients receiving nitroglycerin were compared to patients who had a cesarean delivery at the same gestational age within one day and who did not receive nitroglycerin. Patients who delivered at the MetroHealth System were used as controls, as the standard of care at this system was not to receive nitroglycerin. Maternal and neonatal variables were analyzed using equality of means and Mann-Whitney U tests as appropriate. A p<0.05 was considered statistically significant.

## Results

Twenty-six patients were identified who had cesarean deliveries between 22 weeks and 29 weeks of gestation. They were matched 1:1 with patients delivering within one day of gestation. There were no significant differences between the nitroglycerin (NTG) and control groups for any demographic variables (Table 1). Maternal age, gestational age at delivery, and race/ethnicity among the subjects were not found to be statistically different between groups. Patients receiving NTG had a slightly higher proportion with private insurance than with Medicaid; however, insurance status did not differ statistically between the two groups.

**Table 1:** Demographics for nitroglycerin/non-nitroglycerin groups (n=52).

Outcomes	Groups		p-Value
	Nitro (n=26)	Control (n=26)	
Mean maternal age (SD)	30.7 (6.39)	27.8 (5.97)	0.090
Mean gestational age (weeks) (SD)	26.14 (0.32)	26.14 (0.34)	0.991
Insurance status			
Private insurance, %	34.6	19.2	0.211
Medicaid, %	65.4	80.8	
Race/ethnicity			
White non-Hispanic, %	15.4	30.8	0.504
Black non-Hispanic, %	46.2	46.1	
Hispanic, %	7.7	23.1	
Native-American/Native-Alaskan, %	3.9	0	
Asian, %	11.5	0	
Declined to answer, %	11.5	0	

Our results compared the rates of active segment incisions versus low transverse incisions in the two groups. Active segment incisions included T-incisions, mid-transverse incisions, and classical incisions. The rate of incision within the active segment of the uterus was lower in the group that received intraoperative sublingual NTG (26.9%) compared to the group that did not (61.5%; p=0.012). Quantitative blood loss was significantly lower in the group that received intraoperative nitroglycerin compared to those who did not (583 mL versus 944 mL, respectively; p=0.031) (Table 2).

**Table 2:** Nitroglycerin administration on Apgar (at 1 min and 5 min) and cord pH (n=52).

Outcomes	Groups		p-Value
	Nitro (n=26)	Control (n=26)	
Apgar 1 min <7			
Yes, %	80.77	73.08	0.256
No, %	19.23	26.92	
Apgar 5 min <7			
Yes, %	34.62	19.23	0.211
No, %	65.38	80.77	
Cord pH <7.1			
Yes, %	7.69	15.38	0.385
No, %	92.31	84.62	

There were no major differences in 1-min and 5-min Apgar scores and arterial cord blood pH (Table 3). Post-hoc power analysis, however, determined insufficient power to detect differences in Apgar scores (power=0.16 and 0.34 at p<0.05) and for cord pH (power=0.22 at p<0.05).

**Table 3:** Nitroglycerin administration on incision type and quantitative blood loss (n=52). QBL: quantitative blood loss

Outcomes	Groups		p-Value
	Nitro (n=26)	Control (n=26)	
Incision type			
Active segment, %	26.9	61.5	0.012
Low transverse, %	73.1	38.5	
QBL, mean (SD)	583.04 (40.21) mL	944.27 (158.0) mL	0.031

## Discussion

Our retrospective case-control study showed that intraoperative nitroglycerin administration was associated with a significantly lower rate of active segment uterine incision in patients undergoing cesarean birth and decreased maternal blood loss. Neonatal outcomes were similar between groups, though post-hoc power analysis did show our sample size was not sufficient to show to detect differences in Apgar scores and cord pH.

Our data contribute to the existing literature that nitroglycerin can effectively be used to relax the lower uterine segment to facilitate difficult deliveries. Prior studies have shown the utility of nitroglycerin in obstetrics in the context of uterine relaxation to facilitate difficult fetal extraction, external or internal version, management of retained placenta with a contracted cervix, and management of uterine inversion without apparent maternal adverse effects [7-10]. There is, however, limited data on the usage of nitroglycerin prior to hysterotomy for preterm cesarean delivery. In addition, prior studies have suggested that there may be an increased risk of hemorrhage and uterine extension, which may have limited the use of nitroglycerin during cesarean delivery [9].

In our novel approach, we found that delivering providers with access to sublingual nitroglycerin were more likely to achieve low transverse incision in patients undergoing cesarean delivery in the preterm period, thus decreasing the need for classical or other suboptimal incision types. In addition, contrary to previous concerns that nitroglycerin would increase the rate of maternal hemorrhage due to uterine relaxation, our study demonstrated that the use of nitroglycerin is associated with lower maternal blood loss at delivery. We hypothesize that the lower blood loss is due to the removal of the placenta once the uterus begins to contract and the ability to achieve a low transverse incision into the uterus, which typically has less blood loss than an active segment incision. NTG-exposed neonates had similar Apgar scores and umbilical artery pH in cord blood to controls.

Our study was limited by a small patient population, as several electronic medical record (EMR) changes at Saint Peter's University Hospital prior to 2017 limited our sample size, as medical records prior to 2017 were not accessible. Additionally, the study was conducted at two different institutions, and only one of the institutions used the investigative agent. Therefore, though patients at the institutions were matched in gestational age, confounding differences may exist between the two populations. This study design was needed as the availability of sublingual nitroglycerin became standard of care at one of our hospitals, which precluded the use of controls from that hospital.

## Conclusions

Reducing the rates of active segment incisions during cesarean delivery may decrease overall maternal and fetal morbidity, including hemorrhage, length of maternal hospitalization, fetal outcomes in the index and subsequent pregnancy, and the necessity for repeat cesarean delivery at earlier gestational ages to prevent uterine rupture in subsequent pregnancy. We propose that nitroglycerin should be considered as an adjunctive medication in pregnant persons undergoing preterm cesarean birth, as its use may allow a low transverse incision. These novel data warrant further investigation with prospective studies as well as studies encompassing a larger patient population to study the efficacy and safety of nitroglycerin usage in preterm cesarean deliveries.
